# Supplementary figures and images for: Taxonomic revision of black salamanders of the Aneides flavipunctatus complex (Caudata: Plethodontidae)
Source: PeerJ. 2019 Aug 1;7:e7370. doi: 10.7717/peerj.7370 (PMC6679913; doi:10.7717/peerj.7370)

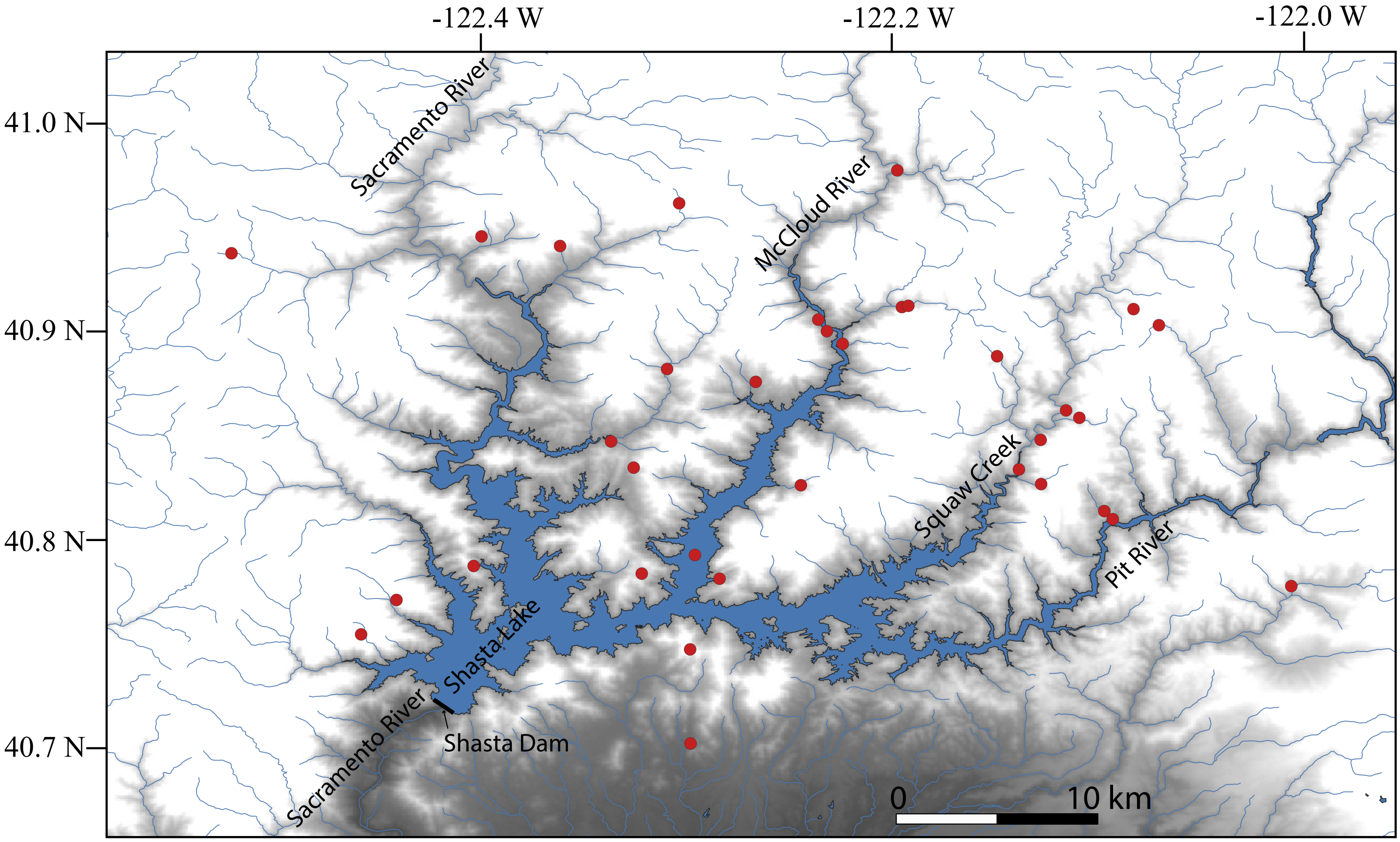

Supplement: Figure S1 — Red dots represent museum localities of Aneides iecanus. Areas below 600 meters are shaded in gray scale and areas above 600 meters are shaded white. [file peerj-07-7370-s002.pdf]
